# The €100 lab: A 3D-printable open-source platform for fluorescence microscopy, optogenetics, and accurate temperature control during behaviour of zebrafish, *Drosophila*, and *Caenorhabditis elegans*

**DOI:** 10.1371/journal.pbio.2002702

**Published:** 2017-07-18

**Authors:** Andre Maia Chagas, Lucia L. Prieto-Godino, Aristides B. Arrenberg, Tom Baden

**Affiliations:** 1 Werner Reichardt Centre for Integrative Neuroscience, University of Tübingen, Tübingen, Germany; 2 Graduate school for Neural and Behavioural Neuroscience, University of Tübingen, Tübingen, Germany; 3 TReND in Africa gUG, Bonn, Germany; 4 Institute of Ophthalmic Research, University of Tübingen, Tübingen, Germany; 5 Center of Integrative Genomics, University of Lausanne, Lausanne, Switzerland; 6 Institute of Neurobiology, University of Tübingen, Tübingen, Germany; 7 School of Life Sciences, University of Sussex, Brighton, United Kingdom

## Abstract

Small, genetically tractable species such as larval zebrafish, *Drosophila*, or *Caenorhabditis elegans* have become key model organisms in modern neuroscience. In addition to their low maintenance costs and easy sharing of strains across labs, one key appeal is the possibility to monitor single or groups of animals in a behavioural arena while controlling the activity of select neurons using optogenetic or thermogenetic tools. However, the purchase of a commercial solution for these types of experiments, including an appropriate camera system as well as a controlled behavioural arena, can be costly. Here, we present a low-cost and modular open-source alternative called ‘FlyPi’. Our design is based on a 3D-printed mainframe, a Raspberry Pi computer, and high-definition camera system as well as Arduino-based optical and thermal control circuits. Depending on the configuration, FlyPi can be assembled for well under €100 and features optional modules for light-emitting diode (LED)-based fluorescence microscopy and optogenetic stimulation as well as a Peltier-based temperature stimulator for thermogenetics. The complete version with all modules costs approximately €200 or substantially less if the user is prepared to ‘shop around’. All functions of FlyPi can be controlled through a custom-written graphical user interface. To demonstrate FlyPi’s capabilities, we present its use in a series of state-of-the-art neurogenetics experiments. In addition, we demonstrate FlyPi’s utility as a medical diagnostic tool as well as a teaching aid at Neurogenetics courses held at several African universities. Taken together, the low cost and modular nature as well as fully open design of FlyPi make it a highly versatile tool in a range of applications, including the classroom, diagnostic centres, and research labs.

## Introduction

The advent of protein engineering has brought about a plethora of genetically encoded actuators and sensors that have revolutionised neuroscience as we knew it but a mere decade ago. On the back of an ever-expanding array of genetically accessible model organisms, these molecular tools have allowed researchers to both monitor and manipulate neuronal processes at unprecedented breadth (e.g., [1–3]). In parallel, developments in consumer-oriented manufacturing techniques such as 3D printing as well as low-cost and user-friendly microelectronic circuits have brought about a silent revolution in the way that individual researchers may customise their lab equipment or build entire setups from scratch (reviewed in: [4–7]). Similarly, already ultra-low-cost light-emitting diodes (LEDs), when collimated, now provide sufficient power to photo-activate most iterations of Channelrhodopsins or excite fluorescent proteins for optical imaging, while a small Peltier-element suffices to thermo-activate heat-sensitive proteins [8–10]. In tandem, falling prices of high-performance charge-coupled device (CCD) chips and optical components such as lenses and spectral filters mean that today, already a basic webcam in combination with coloured, transparent plastic or a diffraction grating may suffice to perform sophisticated optical measurements [11,12]. Taken together, modern biosciences today stand at a precipice of technological possibilities, in which a functional neuroscience laboratory set-up, capable of delivering high-quality data over a wide range of experimental scenarios, can be built from scratch for a mere fraction of the cost traditionally required to purchase any one of its individual components. Here, we present such a design.

Assembled from readily available off-the-shelf mechanical, optical, and electronic components, “FlyPi” provides a modular solution for basic light and fluorescence microscopy as well as time-precise opto- and thermogenetic stimulation during behavioural monitoring of small, genetically tractable model species such as zebrafish (*Danio rerio*), fruit flies *(Drosophila melanogaster)*, or nematodes (e.g., *C*. *elegans*). The system is based on an Arduino microcontroller [13] and a Raspberry Pi 3 single-board computer (RPi3; [14]), which also provides sufficient computing power for basic data analysis, word processing, and web access using a range of fully open-source software solutions that are preinstalled on the secure digital (SD) card image provided. The mechanical chassis is 3D printed, and all source code is open, such that the design and future modifications can be readily distributed electronically to enable rapid sharing across research labs and institutes of science education. This not only facilitates reproducibility of experimental results across labs, but also promotes rapid iteration and prototyping of novel modifications to adapt the basic design for a wide range of specialised applications. More generally, it presents a key step towards a true democratisation of scientific research and education that is largely independent of financial backing [4].

Here, we first present the basic mode of operation, including options for micropositioning of samples and electrodes, and demonstrate FlyPi’s suitability for light microscopy and use as a basic medical diagnostic tool. Second, we present its fluorescence capability including basic calcium imaging using GCaMP5 [1]. Third, we survey FlyPi’s suitability for behavioural tracking of *Drosophila* and *C*. *elegans*. Fourth, we demonstrate optogenetic activation of Channelrhodopsin 2 [3] and CsChrimson [15] in transgenic larval zebrafish as well as *Drosophila* larvae and adults. Fifth, we evaluate the performance of FlyPi’s Peltier-thermistor control loop for thermogenetics [16]. Sixth, we briefly summarise our efforts to introduce this tool for university research and teaching in sub-Saharan Africa [4,17].

## Results

### Overview

**Fig 1:**
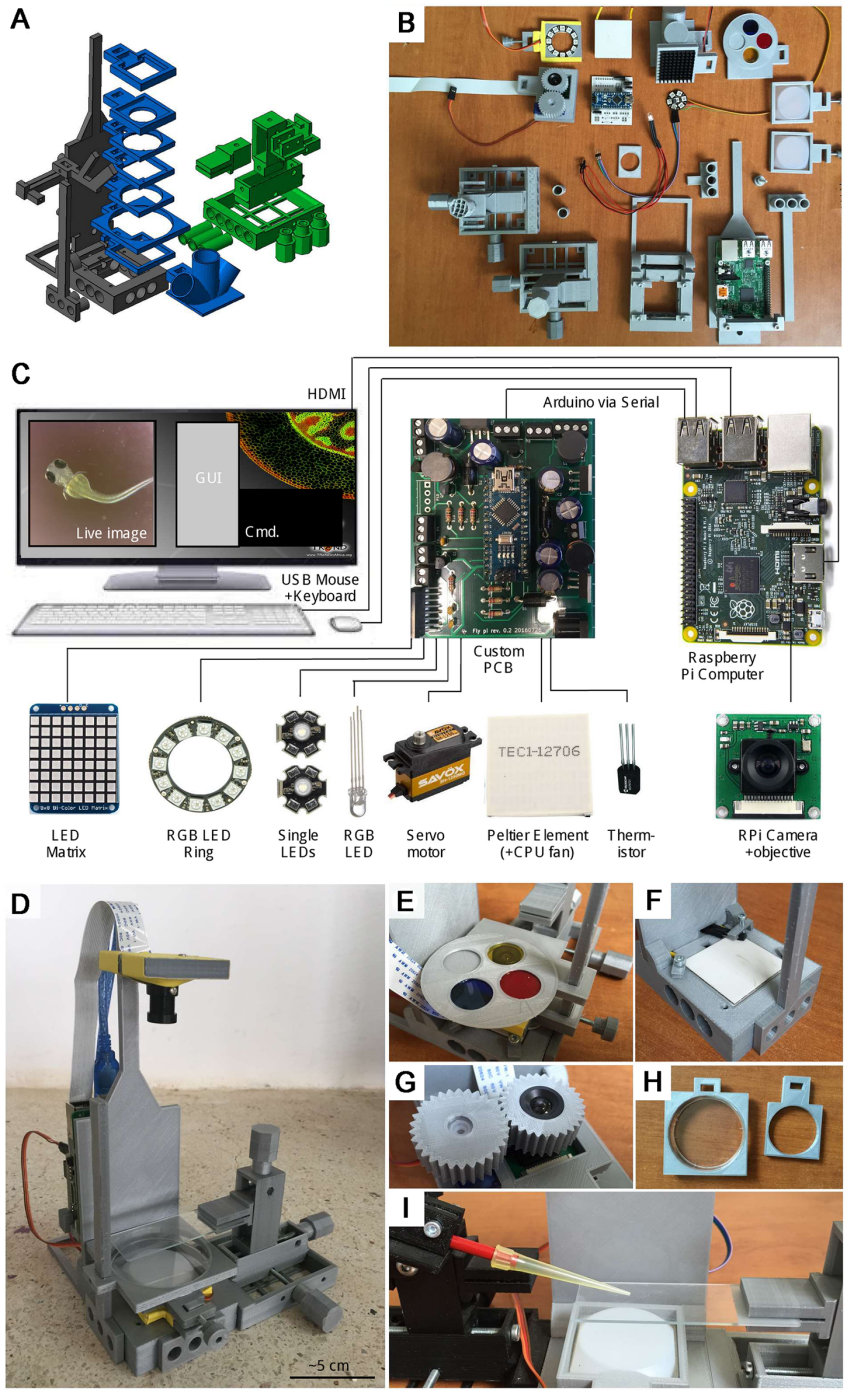
Overview. **A**. The 3D model, colour coded by core structure (black), mounting adapters (blue), and micromanipulator (green). **B**. Printed parts and electronics, partially assembled. **C**. Wiring diagram and summary of electronics. Full bill of materials (BOM) in S1 Table. **D**. The assembled FlyPi with single micromanipulator and light-emitting diode (LED)-ring module, diffusor, and Petri dish adapter mounted in the bottom. **E**. Filter wheel mounted above the inverted camera objective. **F**. Peltier element and thermistor embedded into the base. **G**. Automatic focus drive. **H**. Petri dish mounting adapters. **I**. A second micromanipulator mounted to the left face of FlyPi holding a probe (here, a 200-μl pipette tip for illustration) above the microscope slide mounted by the micromanipulator on the right.

The basic FlyPi can resolve samples down to approximately 10 microns, acquire video at up to 90 Hz, and acquire time-lapse series over many hours. It consists of the 3D-printed mainframe (Fig 1A–1D), one RPi3 computer with a Pi camera and off-the-shelf objective lens, one Arduino-Nano microcontroller, as well as a custom printed circuit board (PCB) for flexible attachment of a wide range of actuators and sensors (Fig 1C). The main printed frame allows modular placement of additional components into the camera path, such as holders for petri dishes or microscope slides (Fig 1D–1I). This basic build, including power adapters, cables, and the module for lighting and optogenetic stimulation, can be assembled for less than €100 (S1 Table; Fig 1D). Additional modules for fluorescence imaging (Fig 1E), temperature control (Fig 1F), or an automated focus drive (Fig 1G) can be added as required. For a full bill of materials (BOM), see S1 Table. A complete user manual and assembly instructions are deposited on GitHub (https://github.com/amchagas/Flypi/blob/master/User%20and%20Assembly%20Manual_revised.pdf). In time, additional content and updated versions will be added to the FlyPi GitHub repository (https://github.com/amchagas/Flypi/).

### Basic camera operation and microscopy

To keep the FlyPi design compact and affordable yet versatile, we made use of the Raspberry Pi (RPi) platform, which offers a range of FlyPi-compatible camera modules. Here, we use the ‘adjustable focus RPi red-green-blue (RGB) camera’ (S1 Table), which includes a powerful 12-mm threaded objective lens. Objective focal distance can be gradually adjusted between approximately 1 mm (peak zoom) and infinity (panoramic, not shown), while the camera delivers 5-megapixel Bayer-filtered colour images at 15 Hz. Spatial binning increases peak framerates to 42 Hz (x2) or 90 Hz (x4). Alternatively, the slightly more expensive 8-megapixel RPi camera or the infrared-capable no-infrared filter (NO-IR) camera can be used. Objective focus can be set manually or via a software-controlled continuous-rotation micro servo motor (Fig 1G). Alternatively, the RPi CCD chip can be directly fitted above any other objective with minimal mechanical adjustments.

A custom-written Graphical User Interface (GUI, S1 Fig) using the Python-based PiCamera library allows for control of framerates, sensitivity, contrast, white balance, and digital zoom. Control over other parameters can be added as required. The GUI facilitates saving images and image sequences in jpeg format and video data in h264 or audio video interleave (AVI) format. Notably, the GUI can also function independent of the remainder of FlyPi components if only easy control for a RPi camera is required.

**Fig 2:**
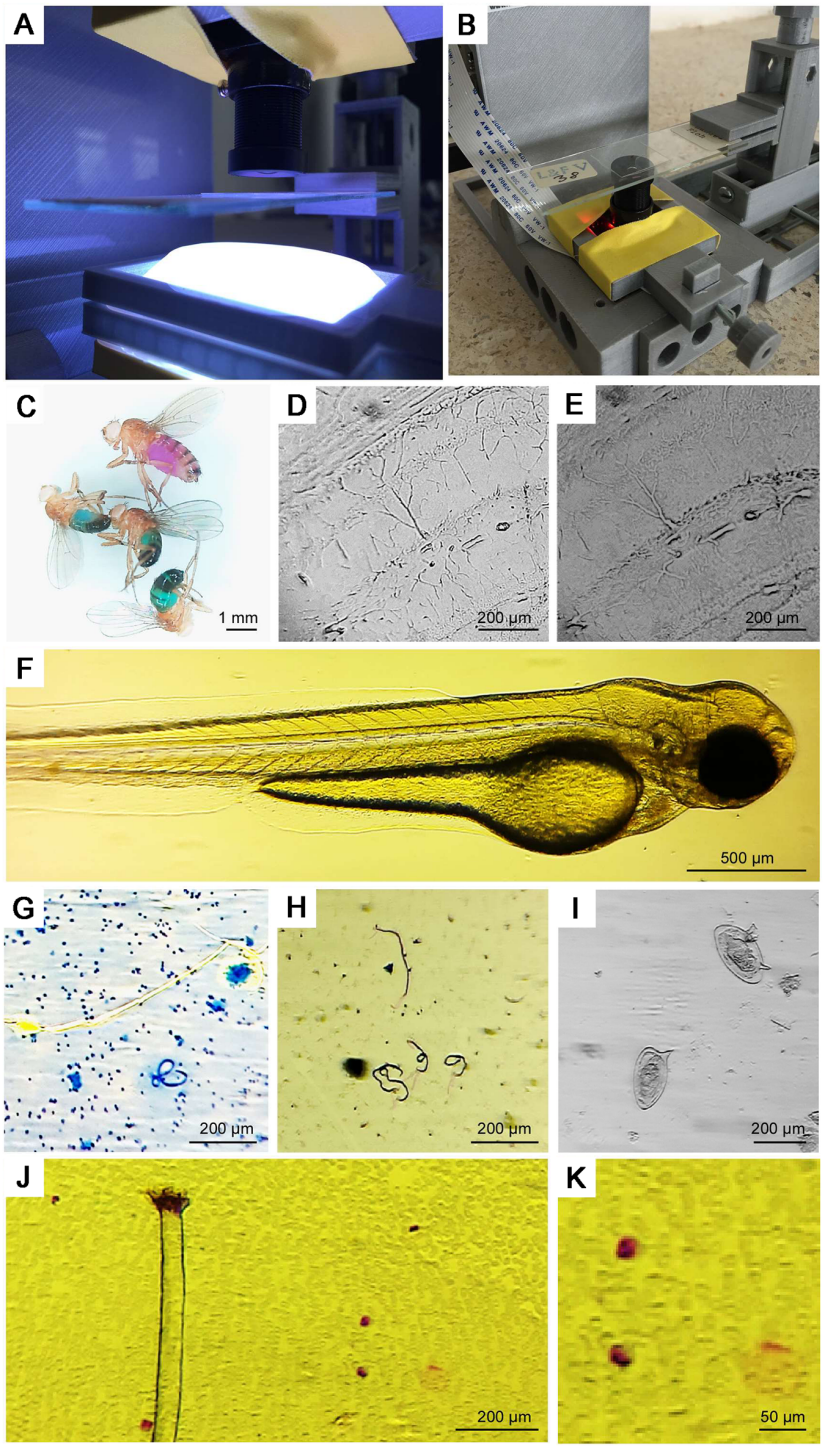
Basic light microscopy. **A**, **B**. The camera and objective can be mounted in upright (A) or inverted mode (B). In each case, the micromanipulator allows accurate positioning of a microscope slide in the image plane, while the light-emitting diode (LED) ring coupled to a series of diffusors provides for flexible spectrum and brightness illumination (A). **C**. At low zoom, the magnification is appropriate to provide high-resolution colour images of several animals at once (here, *D*. *melanogaster* fed with 5 mM sucrose in 0.5% agarose dyed with blue or red food dyes (Food Blue No. 1 and Food Red No. 106 dyes; Tokyo Chemical Industry Co., Japan) as described in [18]. **D**, **E**. When the objective is fully extended, magnification is sufficient to resolve large neurons of the mouse brain, while different positions of the LED ring permit one to highlight different structures in the tissue. **F**. The system is also appropriate to provide high-resolution imagery of zebrafish larvae (*D*. *rerio*) with only room lighting (cf. S1 Video). **G**, **H**. *Brugia malayi* (G) and *Wuchereria bankrofti* (H) in human lymph tissue biopsy. **I**. *Schistosoma* eggs in human urine. **J**. *Mansonella perstans* in human blood smear (Wright Giemsa stain) and **K**. magnification of bottom-right image section.

The camera can be mounted in two main configurations: upright or inverted (Fig 2A and 2B). While the former may be primarily used for resolving larger objects such as adult *Drosophila* (Fig 2C) or for behavioural tracking, the latter may be preferred for higher-zoom applications (Fig 2D and 2E) and fluorescence microscopy (see below) or if easy access to the top of a sample is required. Here, the image quality is easily sufficient to monitor basic physiological processes such as the heartbeat or blood flow in live zebrafish larvae (Fig 2F, S1 Video).

If required, specimens can be positioned by a 3D-printed micromanipulator [4] (Fig 2B). Up to three manipulators can be attached to the free faces of FlyPi (Fig 1D and 1I). Manipulators can also be configured to hold probes such as electrodes or stimulation devices (Fig 1I). Like the camera objective, manipulators can be optionally fitted with continuous-rotation servo motors to provide electronic control of movement in three axes [4]. These motors can be either controlled via software or via a stand-alone joystick unit based on a separate Arduino-Uno microcontroller and a Sparkfun Joystick shield [19]. Depending on print quality and manipulator configuration, precision is in the order of tens of microns [4].

For lighting, we use an Adafruit Neopixel 12 LED ring [20] comprising 12 high-power RGB-LEDs that can be configured for flexible intensity and wavelength lighting. For example, the LED ring with all LEDs active simultaneously can be used to add ‘white’ incident or transmission illumination (e.g., Fig 2A and 2F) while behavioural tracking can be performed under dim, red light. A series of white weighing boats mounted above the ring can be used as diffusors (Fig 2A). Long-term time-lapse imaging, for example, to monitor developmental processes or bacterial growth, can be performed in any configuration. Lighting is controlled from the GUI through an open Adafruit LED control Python library.

The implementation of a cost-effective option for digital microscopy also opens up possibilities for basic medical diagnosis, such as the detection of small parasitic nematodes *Brugia malayi* or *Wuchereria bankrofti* in human lymph tissue samples (Fig 2G and 2H) or *Schistosoma* eggs in human urine (Fig 2I). Similarly, the image is sufficient to detect and identify counterstained types of blood cells in an infected smear (here, *Mansonella perstans*; Fig 2J and 2K).

### Fluorescence microscopy

**Fig 3:**
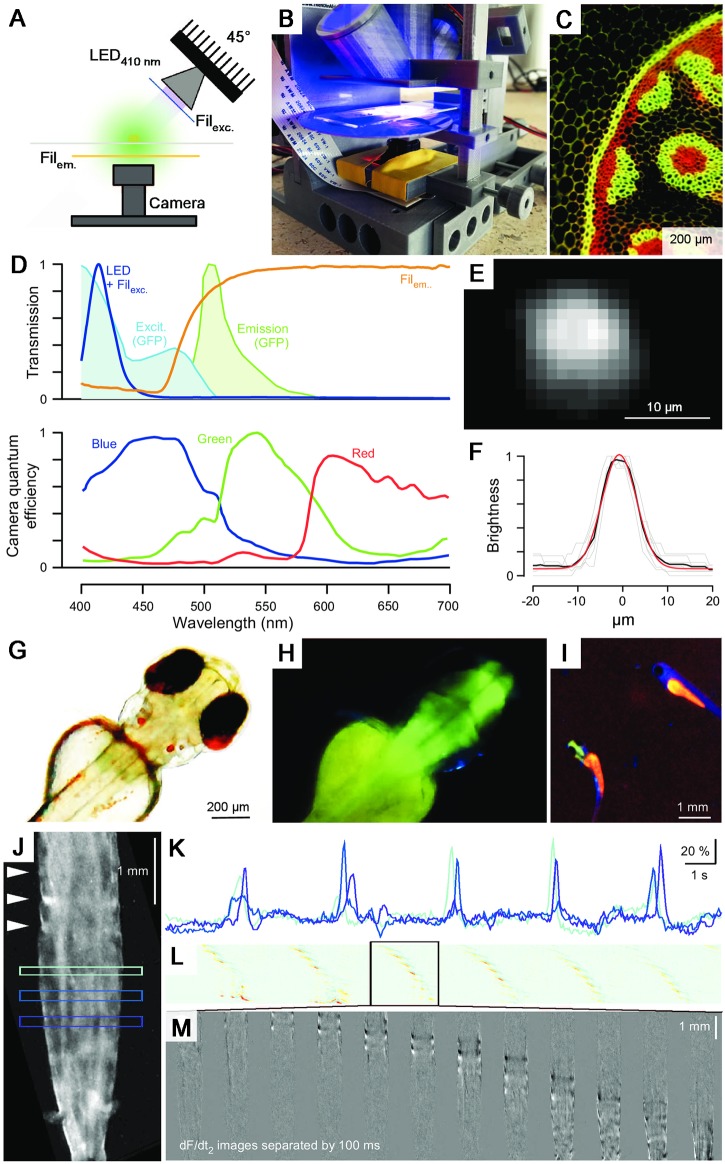
Fluorescence microscopy. **A**. A collimated 410 nm light-emitting diode (LED) angled at 45° and two ultra-low-cost theatre lighting filters provide for fluorescence capability. **B**. A photo of the fluorescence setup. **C**. Fluorescence test slide. **D**. Top: Spectra of excitation LED and filters superimposed (dark blue) on green fluorescent protein (GFP) excitation (light blue) and emission (green) spectra. Emission filter in orange. Bottom: Quantum efficiency of the OmniVision OV5647 charge-coupled device (CCD) camera chip used in the Raspberry Pi (RPi) system, taken from [23]. **E**, **F**. Point-spread function (*psf*) measured using green fluorescent beads (Methods): Standard deviation is approximately 5.4 μm. **G**, **H**. Three days postfertilisation (*dpf*) Zebrafish larva expressing GCaMP5Gf in neurons (*HuC*:*GCaMP5G*) in transmission (G) and fluorescence mode (H). **I**. At low zoom, the system can be used for fish sorting (cf. S2 Video). Note the absence of green fluorescence in the brain of the nontransgenic animal to the upper right. **J-M**. Calcium Imaging in *Drosophila* larva expressing GCaMP5 in muscles (Mef2-Gal4; UAS-myr::GCaMP5). **J**, **K**. Three regions of interest placed across the raw image stack of a freely crawling larva (J) reveal period bouts of increased fluorescence, as peristaltic waves drive up calcium in muscles along the body (K). Arrowheads in J indicate the positions of peaks in a calcium wave. **L**. A space–time plot of the time-differentiated image stack, averaged across the short body axis, reveals regular peristaltic waves. Warm colours indicate high positive rates of change in local image brightness. **M**. A single peristaltic wave (as indicated in L) in 12 image planes separated by 100-ms intervals (cf. S5 Video).

Next, we implemented fluorescence capability based on a 350 mA 410 nm LED attached to a reflective collimator as well as ultra-low-cost theatre lighting filters. For this, the excitation and emission light was limited by a low-pass and a notch filter, respectively (Fig 3A–3D, S1 Table). Imperfect emission filter efficiency for blocking direct excitation light necessitated that the source was positioned at 45° relative to the objective plane, thereby preventing direct excitation bleed-through into the camera path (Fig 3A and 3B). Many commonly used fluorescent proteins and synthetic probes exhibit multiple excitation peaks. For example, Green Fluorescent Protein (GFP) is traditionally excited around 488 nm; however, there is a second and larger excitation peak in the near UV [21] (Fig 3D, but see [1]). Here, we made use of this short-wavelength peak by stimulating at 410 nm to improve spectral separation of excitation and emission light despite the suboptimal emission filter. Fig 3C shows the fluorescence image recorded in a typical fluorescence test slide. The RGB camera chip allowed simultaneous visualisation of both green and red emission. If required, the red channel could be limited either through image processing or by addition of an appropriate short-pass emission filter positioned above the camera. Next, using green fluorescent beads (100 nm, Methods), we measured the point-spread function (*psf*) of the objective as 5.4 μm (s.d.) at full zoom (Fig 3E and 3F). This is >10 times broader than that of a typical state-of-the-art confocal or 2-photon system [22], though without optical sectioning, and imposes a theoretical resolution limit in the order of approximately 10 μm. Notably, with an effective pixel size of approximately 1 μm (Fig 1E), the system is therefore limited by the objective optics rather than the resolution of the camera chip, such that the use of a higher numerical aperture objective would yield a substantial improvement in spatial resolution. It also means that at peak zoom, the camera image can be binned at x4 for increased speed and sensitivity without substantial loss in image quality.

Next, we tested FlyPi’s performance during fluorescence imaging on live animals. At lower magnification, image quality was sufficient for basic fluorescence detection as required, for example, for fluorescence-based sorting of transgenic animals (screening). We illustrate this using a transgenic zebrafish larva (3 days postfertilisation [*dpf*]) expressing the GFP-based calcium sensor GCaMP5G in all neurons (Fig 3G–3I, S2 Video). Similarly, the system also provided a sufficient signal-to-noise ratio for basic calcium imaging, demonstrated here using *Drosophila* larvae driving GCaMP5 in muscles that reveal clear fluorescence signals associated with peristaltic waves as the animal freely crawls on a microscope slide (Fig 3J–3M; see also S3 Video, cf. [24]). However, the system failed to provide a sufficient signal-to-noise ratio for imaging clear calcium signals in substantially smaller structures, such as neurons of the *Drosophila* antennal lobe or zebrafish optic tectum (not shown). This is likely related to the limited optical power and large field of depth of the objective used, and might, therefore, be ameliorated in the future by integration of different commercial objectives and/or optical filters. Further fluorescence example videos are provided in the supplementary materials (S4 and S5 Videos).

### Behavioural tracking

**Fig 4:**
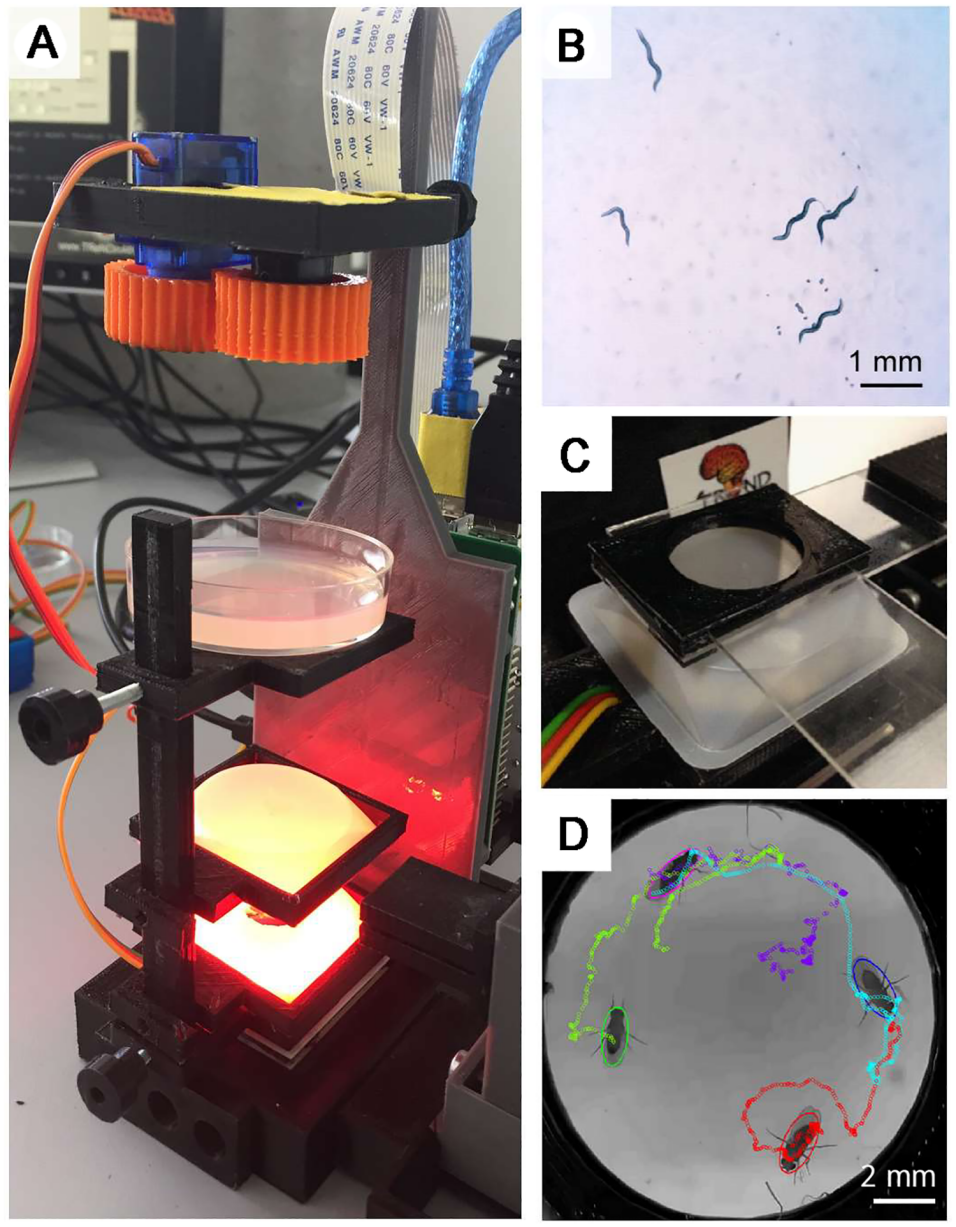
Behavioural tracking. **A**, **B**. Red light illumination from the light-emitting diode (LED) ring can be used to illuminate animals during behavioural tracking—*C*. *elegans* is shown here on an Agar plate (B). **C**. A behavioural chamber based on two microscope slides and a 3D-printed chassis is adequate for behavioural monitoring of adult *Drosophila*. **D**. Animals tracked using Ctrax [25].

‘To move is all mankind can do’. Sherrington’s (1924) thoughts on the ultimate role of any animal’s nervous system still echoes today, when despite decades of (bio)technological advances, behavioural experiments are still amongst the most powerful means for understanding neuronal function and organisation. Typically, individual or groups of animals are placed in a controlled environment and filmed using a camera system. Here, FlyPi’s colour camera with adjustable zoom offers a wide range of video-monitoring options, while the RGB LED ring provides for easily adjusted wavelength and intensity lighting (Fig 4A) including dim red light, which is largely invisible to many invertebrates including *C*. *elegans* (Fig 4B, S6 Video) and *Drosophila*. A series of mounting adapters for petri dishes (Fig 1H) as well as a custom chamber consisting of a 3D-printed chassis and two glass microscope slides for adult *Drosophila* (Fig 4C) can be used as behavioural arenas. Following data acquisition, videos are typically fed through a series of tracking and annotation routines to note the spatial position, orientation, or behavioural patterns of each animal. Today, a vast range of open behavioural analysis packages is available, including many that run directly on the RPi3 such as CTrax [25], used here to track the movements of adult *Drosophila* in a 10-s video (Fig 4D; S7 Video).

### Optogenetics and thermogenetics

One key advantage of using genetically tractable model organisms is the ability to selectively express proteins in select populations of cells whose state can be precisely controlled using external physical stimuli such as light (Optogenetic effectors, e.g., [26]) or heat (Thermogenetic effectors, e.g., [16]). Through these, the function of individual or sets of neurons can be readily studied in behavioural experiments. A plethora of both light- and heat- sensitive proteins are available, with new variants being continuously developed. Many of these proteins exhibit sufficient sensitivity for activation by collimated high-power LEDs, rather than having to rely on more expensive light sources like a Xenon lamp or a laser. Similarly, temperature variation over a few degrees Celsius, as achieved by an off-the-shelf Peltier element with adequate heat dissipation, is sufficient to activate or inactivate a range of temperature-sensitive proteins. We therefore implemented both opto- and thermogenetic stimulation capability for FlyPi.

#### Optogenetics

**Fig 5:**
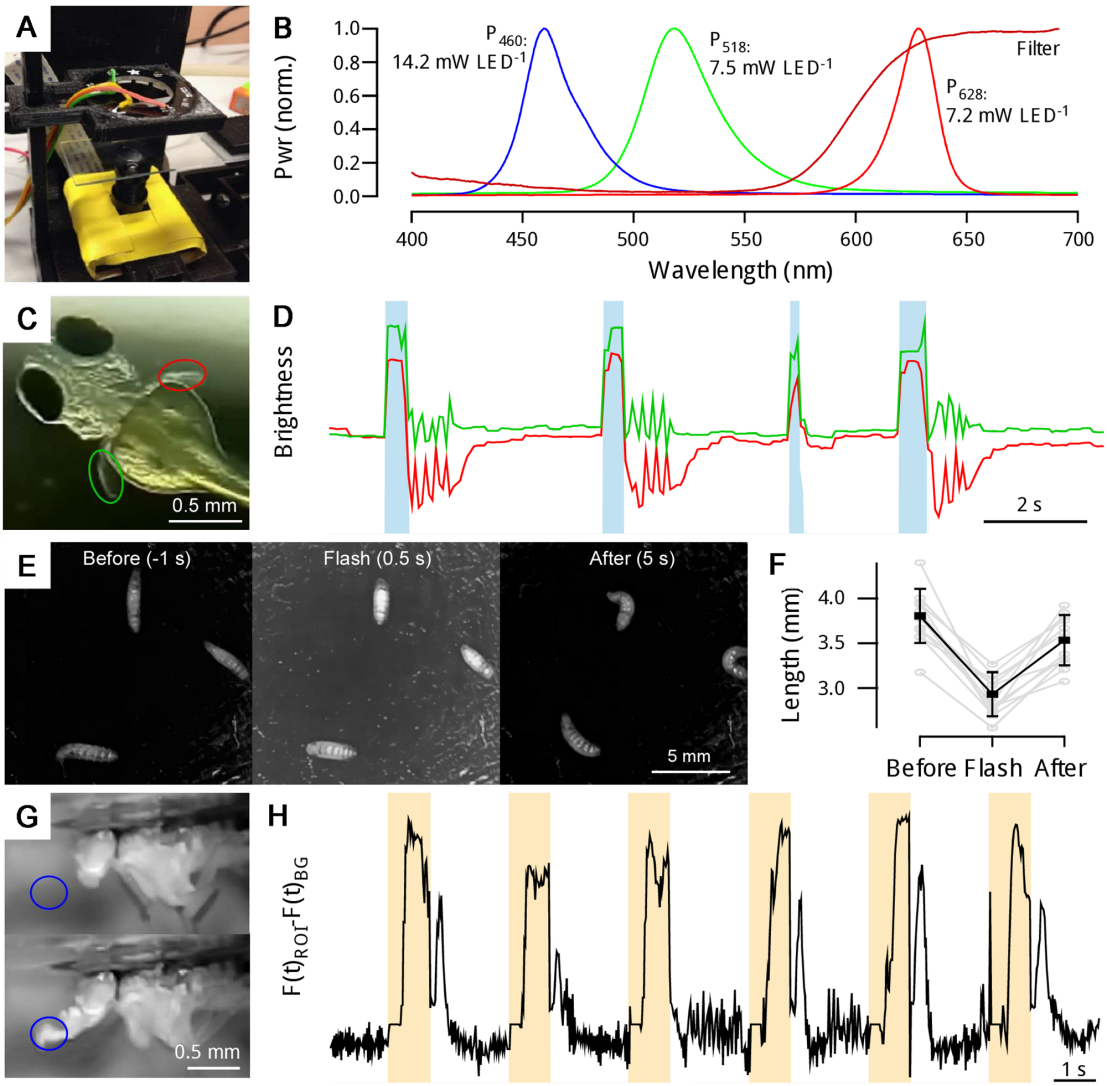
Optogenetics. **A**. Experimental configuration suitable for optogenetic stimulation of an individual zebrafish larva suspended in a drop of E3 (Methods). **B**. Spectrum and peak power of the three light-emitting diodes (LEDs) embedded at each ring position. Spectral filters can be used to limit excitation light reaching the camera (Rosco Supergel No. 19, ‘Fire’). **C**. Zebrafish larva (3 days postfertilisation [*dpf*]) expressing ChR2 broadly in neurons (*Et(E1b*:*Gal4)s1101t*, *Tg(UAS*::*Cr*.*ChR2_H134R-mCherry)s1985t*, *nacre-/-*). **D**. The animal exhibits pectoral fin burst motor patterns upon activation of blue LEDs (cf. S8 Video). **E, F**. *Drosophila* larvae expressing ChR2 in all neurons (elav-GAL4/+; UAS-shibre^ts^; UAS-ChR2/+; UAS-ChR2/+) crawling on ink-stained agar reliably contract when blue LEDs are active. **G**, **H**. Proboscis extension reflex (PER) in adult *Drosophila* expressing CsChrimson in the gustatory circuit (w; +; GMR86A08-GAL4/UAS-CSChrimson; the GMR86A08-GAL4 is part of the Janelia Farm Flightlight collection [32]; its effect on PER is a personal communication from Olivia Schwarz and Jan Pielage, University of Kaiserslautern, Germany, who observed this phenotype as part of behavioural screen [33]) is reliably elicited by activation of red LEDs.

For optogenetic activation, we used the LED ring (Fig 5A), whose spectrum and power are appropriate for use with both ChR2 (single LED ‘blue’ Pwr_460_: 14.2 mW) as well as ReaChr and CsChrimson (‘red’ Pwr_628_: 7.2 mW; ‘green’ Pwr_518_: 7.5 mW) (Fig 5B) [3,15,27,28]. Alternatively, an Adafruit 8 x 8 high-power single-wavelength LED matrix [20] can be attached for spatially selective optogenetic or visual stimulation [29]. For demonstration, a zebrafish larva (3 *dpf*) expressing ChR2 in all neurons was mounted on top of a microscope slide, which was in turn held above the inverted objective using the micromanipulator (Fig 5A and 5C). The LED ring was positioned facedown approximately 2 cm above the animal, outside of the centrally positioned camera’s field of view. Concurrent maximal activation of all 12 ‘blue’ LEDs (Pwr_460_: approximately 4.9 mW cm^−2^ at the level of the specimen) reliably elicited basic motor patterns for stimuli exceeding 500 ms, illustrated here by pectoral fin swimming bouts (Fig 5C and 5D, S8 Video). Substantially shorter stimuli did not elicit the behaviour (e.g., third trial: approximately 150 ms), nor did activation of the other wavelength LEDs or blue light activation in ChR2-negative control animals (not shown). This strongly indicated that motor networks were activated through ChR2 rather than innate, visually mediated escape reflexes in response to the light (cf. [30]) or photomotor responses [31]. Notably, in the example shown, while the stimulus artefact was used as a timing marker, excitation light could be blocked (>95% attenuation) using an appropriate filter (Fig 5B dark red trace, S1 Table) without substantially affecting image quality, while timing could be verified using the flexibly programmable low-power RGB LED normally integrated into the Peltier-thermistor loop (not shown).

We also tested ChR2 activation in *Drosophila* larvae. Animals were left to freely crawl on ink-stained agarose with both the LED ring and camera positioned above. Activation of all 12 blue LEDs reliably triggered body contractions for the duration of the 1-s stimulus, followed by rapid recovery (Fig 5E and 5F). Finally, full-power activation of the red LEDs reliably triggered proboscis extension reflex (PER) in adult *Drosophila* expressing CsChrimson in the gustatory circuit (Fig 5G and 5H). In this latter demonstration, we made use of the GUI’s protocol function, which allows easy programming of microsecond-precision looping patterns controlling key FlyPi functions such as LEDs and the Peltier Loop (see below).

#### Thermogenetics

**Fig 6:**
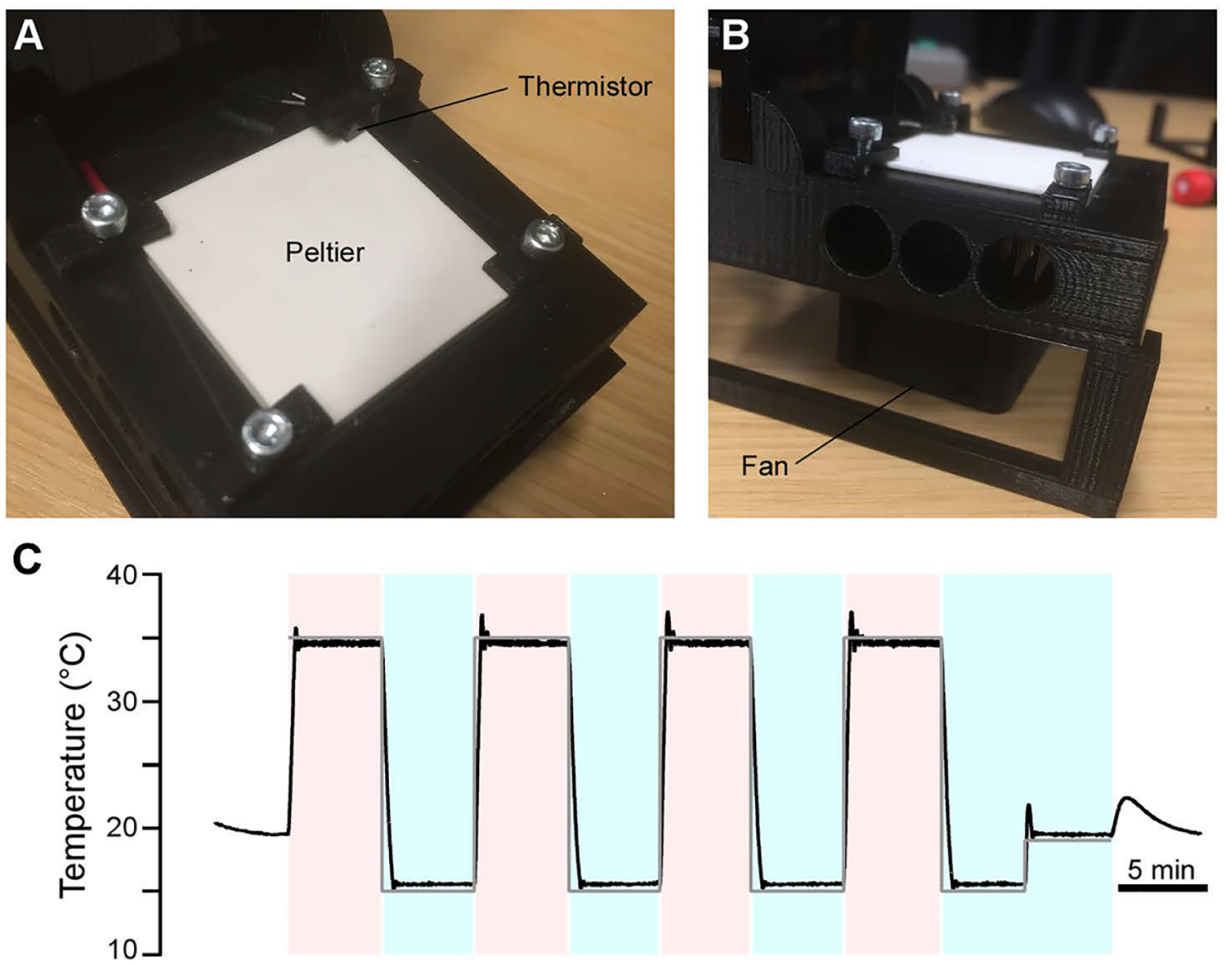
Thermogenetics. **A**. The 4 cm x 4 cm Peltier element embedded in the FlyPi base, with the Thermistor clamped into one corner. **B**. Side view with FlyPi propped up on a set of 3D-printed feet to allow air dissipation beneath the base. The central processing unit (CPU) fan is positioned directly beneath the Peltier. **C**. Performance of the Peltier-thermistor feedback loop. Command 15°C and 35°C indicated by blue and red shading, switching every 5 minutes; room temperature 19°C (no shading).

Owing to their remarkable ability to tolerate a wide range of ambient temperatures, many invertebrate model species including *Drosophila* and *C*. *elegans* also lend themselves to thermogenetic manipulation. Through the select expression of proteins such as Trp-A or shibire^ts^ [16,34], sets of neurons can be readily activated or have their synaptic drive blocked by raising the ambient temperature over a narrow threshold of 28°C and 32°C, respectively. Here, FlyPi offers the possibility to accurately control the temperature of the upper surface of a 4 cm x 4 cm Peltier element embedded in its base, with immediate feedback from a temperature sensor (Fig 6A, S1 Table). A central processing unit (CPU) fan and heat sink below the Peltier element dissipate excess heat (Fig 6B, S1 Table). The setup reaches surface temperatures ± approximately 20°C around ambient temperature within seconds (approximately 1°C/s) and holds set temperatures steady over many minutes (s.d. < 1°C) (Fig 6C).

## Discussion

We primarily designed FlyPi to achieve a good balance of performance, cost, and flexibility in its use. Using higher quality components, individual function performance can certainly be improved (see Potential for further development). Here, it is instructive to compare FlyPi’s microscope function to other open microscope designs. For example, the fully 3D-printable microscope stage of the “Waterscope” [35] achieves superior stability of the focussing mechanism. However, unlike FlyPi, this design cannot achieve the same range of possible magnifications needed for behavioural experiments. Some other open microscope designs (e.g., [36,37]) use a larger fraction of commercial components to provide superior image quality and/or stability, albeit invariably at a substantially higher cost. On the extreme low-cost scale, available designs typically do not provide the imaging systems (i.e., the camera, control software, and processor) but instead rely on the addition of a mobile phone camera or, indeed, the eye itself (e.g., [38–40]). Next, FlyPi also provides for a powerful range of sample illumination options, which typically exceed available alternatives. To our knowledge, no alternative open microscope design encompasses the experimental accessories and control systems required for behavioural tracking under both opto- and thermogenetic control.

Another key aspect of FlyPi’s design is its modular nature. This means that the system does not require all integrated options to be assembled to function. For example, if the main purpose of an assembled unit is to excite Channelrhodopsin, the only module beside the base unit is the LED ring. Similarly, only the Peltier-thermistor circuit is needed for Thermogenetics experiments. This means that units designed for a dedicated purpose can be assembled quickly and at substantially reduced cost. Moreover, given a functional base unit, it is easy for the user to modify any one part or to integrate a fully independent module built for a different purpose altogether. The modular nature also renders the design more robust in the face of difficulties with sourcing building components.

### Potential for further development

Clearly, the current FlyPi only scratches the surface of possible applications. Further development is expected to take place as researchers and educators integrate aspects of our design into their laboratory routines. To explicitly encourage resharing of such designs with the community, we maintain and curate a centralised official project page (http://open-labware.net/projects/flypi/) linked to a code repository (https://github.com/amchagas/Flypi). Indeed, a basic description of the FlyPi project has been online since 2015, which has led to several community-driven modifications. For example, a recent modification of the 3D-printed mainframe implements the camera and focus motor below a closed stage [41]. At the expense of a fixed camera position, this build is substantially more robust and thus perhaps more suitable, e.g., for classroom teaching. Other community-driven modifications include a version in which all 3D-printed parts are replaced by Lego^®^ blocks [42] as well as several forks geared to optimise the code, details in the 3D model, or additions in the electronic control circuits.

Currently, one obvious limit of FlyPi is spatial resolution. The system currently resolves individual human red blood cells (Fig 2K), but narrowly fails to resolve malaria parasites within (not shown). Here, the limit is optical rather than related to the camera chip, meaning that use of a higher numerical aperture and magnification objective lens will yield substantial improvements. This development might come in hand with additional improvements in the micromanipulator’s Z-axis stability to facilitate focussing at higher magnification—for example, as implemented in the Waterscope [35]. Similarly, photon catch efficiency of the CCD sensor could be improved by use of an unfiltered (monochrome) chip. Unfortunately, to date, no such chip is available for the RPi platform; however, it may be possible to carefully scratch off the Bayer filter using a wooden chisel [43]. Next, the contrast of fluorescence images could be markedly improved by further investment in optical filters. Other alleys of potential further development include the following: (i) the addition of further options for fluorescence microscopy to work over a wider range of wavelengths, likely through the use of other excitation LEDs and spectral filters; (ii) FlyPi could also be tested for stimulating photo-conversion of genetically encoded proteins such as CamPari, Kaede, or photoconvertible GFP [44–46]; (iii) auto-focussing could be implemented by iteratively rotating the servo-assisted focus while evaluating changes in the spatial autocorrelation function or Fourier spectrum of the live image; (iv) a motorised manipulator could be integrated for stage-automation through a simple software routine; and (v) one or several FlyPis could be networked wirelessly or through the integrated Ethernet port to allow centralised access and control, thereby removing the need for dedicated user interface peripherals. Taken together, by providing all source code and designs under an open-source license, together with an expandable online repository, we aim to provide a flexible, modular platform upon which enthusiastic colleagues may build and exchange modifications in time. We will be pleased to add modifications to our basic design to the online project repositories as appropriate. For this, please contact the first author directly.

### Classroom teaching and laboratory improvisation

In large parts of the world, funding restrictions hamper the widespread implementation of practical science education—a problem that is pervasive across both schools and universities [4,47]. Often, limitations include broken or complete lack of basic equipment such as low-power light microscopes or computing resources. Here, the low cost and robustness of FlyPi may offer a viable solution. If only one unit can be made available for an entire classroom, the teacher can project the display output of FlyPi to the wall such that many students can follow demonstrated experiments. Already a low amount of funding may furnish an entire classroom with FlyPis, allowing students in groups of two or three to work on and maintain their own unit. The relative ease of assembly also means that building FlyPi itself could be integrated into part of the syllabus. In this way, a basic technical education in electronics and soldering or basic 3D printing could be conveyed in parallel. As an additional advantage, each student could build their own equipment, which brings about further benefits in equipment maintenance and long-term use beyond the classroom.

To survey to what extent FlyPi assembly and use may be beneficial in a classroom scenario, we introduced the equipment to African biomedical MSc and PhD students as well as senior members of faculty during a series of multi-day workshops at Universities in sub-Saharan Africa since 2015, including the University of KwaZulu Natal (Durban, SA), the International Centre of Insect Physiology and Ecology (icipe, Nairobi, Kenya), Kampala International University (Dar es Salaam, Tanzania and Ishaka Bushyeni, Uganda), and the International Medical and Technical University (IMTU, Dar es Salaam, Tanzania). In addition, colleagues have used and modified the design for projects held in Accra, Ghana, Khartoum, Sudan, and Ishaka, Uganda. In one workshop, we only provided the 3D-printed parts, the custom PCB, and off-the-shelf electronics and took students though the entire process of assembly and installation. Having had no previous experience with basic electronics, soldering, or the use of simple hand tools such as a Dremel or cable-strippers, all students successfully assembled a working unit. Towards the end of the training, students used their own FlyPi to perform basic neurogenetics experiments with *Drosophila*, including heat activation of larvae expressing shibire^ts^ in all neurons (elav-GAL4/+; UAS-shibre^ts^,UAS-ChR2 / +; UAS-ChR2 / +, cf. Fig 6) and optogenetic activation of ChR2 to elicit a range of behaviours in both adults and larvae (cf. Fig 5). Following the training, students took their assembled FlyPis home for their own research and teaching purposes. In other courses, we brought preassembled FlyPis with a range of different modules. Students learned to operate the equipment within minutes and subsequently used them for a range of experiments and microscopy tasks, including several novel configurations not formally introduced by the faculty. Indeed, many experiments presented in this paper were performed during these training courses. We also used individual FlyPi modules to improvise workarounds for incomplete commercial lab equipment. For example, the RPi camera with focus drive and live image-processing options served as an excellent replacement for a missing Gel-doc camera. Similarly, we used FlyPi as a replacement camera for odour-evoked calcium imaging in *Drosophila* antennas on a commercial, upright fluorescence microscope or for dissection demonstrations under a stereoscope that also utilised the LED rings for illumination. Moreover, FlyPi’s programmable General Purpose Pins (GPPs) and LEDs were used to drive time-precise light-stimulus series, e.g., for independently recorded *Drosophila* electroretinograms (ERGs). Similarly, the Peltier-feedback circuit was adequate to maintain developing zebrafish embryos at a controlled temperature during prolonged experiments or to reversibly block action potential propagation in long nerves through local cooling. Clearly, beyond its use as a self-standing piece of equipment and teaching tool, the low cost and modular nature of FlyPi also renders it versatile to support or take over a wide range of additional functions in the lab. In hand, the use and assembly of a system like FlyPi may inspire confidence in researchers’ ability to build and modify other pieces of equipment themselves, shaped to their individual needs, which in a teaching scenario is perhaps the greatest benefit of all.

## Conclusion

Taken together, we anticipate that the open design of FlyPi will be useful in scientific teaching and research as well as for medical professionals working in low-resource settings looking to supplement their diagnostic toolkit. We anticipate that in time, further improvement and new designs will emerge from the global open hardware community. Notably, a curated collection of further “Open-Labware” [4,48] designs can be found on the PLOS website [49].

## Methods

A complete assembly and user manual is deposited on GitHub (https://github.com/amchagas/Flypi/blob/master/User%20and%20Assembly%20Manual_revised.pdf).

### Assembly time and necessary skills

From our previous workshop experiences, the assembly of the FlyPi (including software setup) should take about 5 hours for a person with no previous soldering experience, provided that all individual components are in place. Experienced users can expect to be done in 2 to 3 hours.

### 3D modelling and printing

3D modelling was performed in OpenSCAD [50], and all files are provided as both editable scad and complied surface tesselation lattice (stl) files. All parts were printed in polylactic acid (PLA) on an Ultimaker 2 3D printer (Ultimaker, Geldermailsen, Netherlands) in six prearranged plates using the following parameters: infill 30%, no supports, 5-mm brim, layer height 0.1 mm, print speed 60 mm/s, and travel speed 200 mm/s. Total printing time of a single FlyPi, including all presented modules, was about 40 hours. Notably, this time can be substantially reduced by using faster print settings and/or a larger nozzle, as is commonly implemented in lower-cost 3D printers. For example, using a well-calibrated delta Rep-Rap delta (www.reprap.org) printing at full speed, the entire system can be printed at sufficient precision in less than 20 hours. In case a 3D printer is not locally available, several available “print-on-demand” services (e.g., Shapeways, Sculpteo, 3D hubs) can be used to source the parts. We estimated that the cost to have parts printed in plastic from one of the services available at 3Dhubs to be approximately €40.

### PCB design and printing

The PCB was designed in KiCad [51] and is provided as the native KiCad file format, as well as the more widely used gerber file format. The PCB facilitates connections between peripherals and the microcontroller and was designed to be modular such that only components that will be used need to be soldered on the board. The power circuitry designed for a single 12 V 5 A power supply is provided. The large spacing between component slots, PCB labelling, and consistent use of the “through-hole” component format is intended to facilitate assembly by users with little soldering experience. Using the provided Gerber files, it is possible to order the PCBs from a variety of producers (e.g., pcbway.com, seeedstudio.com/pcb, dirtypcbs.com). Of course, if required, the entire PCB could also be improvised using individual cables and/or a suitable breadboard by taking reference to the circuit diagram provided.

### The GUI

The GUI (S1 Fig) was written in Python3. The control functions for each peripheral component is created in its own class, making it easier for the end user to create/alter functions independently. These classes are then contained in a ‘general purpose’ class, responsible for the display of the user interface and addressing the commands to be sent to the Arduino board (responsible for time-precise events and direct interaction with peripherals, for details see below). The communication between the RPi and the Arduino is established via universal serial bus (USB) through a serial protocol (Python Serial library [52]). The GUI is created using Tkinter [53]. Both libraries are compatible with Python2 and Python3.

The GUI is also capable of creating folders and saving files to the Raspberry Pi desktop. For simplicity, the software creates a folder called ‘FlyPi_output’ and subfolders depending on the type of data being acquired (time lapse, video, snapshots, temperature logging). The files within the subfolders are created using date and time as their names, preventing overwriting of data.

### Arduino

We used an ATmega328-based Arduino Nano [13]. The board was chosen due to a high number of input/output ports, its variety of communication protocols (e.g., Serial, I2C), its low cost and easy availability (including several ultra-low-cost clones at €2 to €3), very well-documented environment (hardware specifications, function descriptions, ‘how to’ recipes), and large user database. The board is programmed in C++ together with the modifications added by the Arduino integrated development environment (IDE). The board is responsible for controlling all peripheral devices except the camera and provides microsecond precision for time measurement. The code can be adapted to most of the other boards of the Arduino family, with small changes (e.g., digital, analogue, and serial port addresses).

### Raspberry Pi 3 operating system

We used Raspian [54] as the operating system (OS) on the Raspberry Pi 3 [14] for its installation simplicity through ‘new out of the box software’ (NOOBS) [55] and because it is derived from Debian [56], a stable and well-supported GNU-Linux distribution. However, any Linux distribution compatible with the Raspberry Pi and the chosen Python3 libraries can be used. Arduino compatibility is not mandatory, since once the board is loaded with the correct code, which can be done on any computer, the Arduino IDE is not used further, as all live communication goes via the serial port directly from Python.

### Spectral and power measurements

We used a commercial photo-spectrometer (USB2000+VIS-NIR, Ocean Optics, Ostfildern, Germany) and custom-written software in Igor-Pro 7 (Wavemetrics) to record and analyse spectra of LEDs and filters. Peak LED power was determined using a Powermeter (Model 818, 200–1800 nm, Newport). We used fluorescent beads (PS-Speck TM Microscope Point Source Kit P-7220, ThermoFisher) for estimating FlyPi’s *psf*.

### Video and image acquisition

All static image data was obtained as full-resolution RGB images (2592 x 1944 pixels) and saved as jpeg. All video data was obtained as RGB at 42 Hz (x2 binning), yielding image stack of 1296 x 972 pixels, and saved as h264. Video data was converted to AVI using the ffmpeg package for GNU/Linux (ffmpeg.org, a conversion button is added to the GUI for simplicity). All further data analysis was performed in Image-J (NIH) and Igor-Pro 7 (Wavemetrics). Figures were prepared in Canvas 15 (ACD Systems).

### Calcium imaging in larval *Drosophila* muscles

Second instar larvae (Mef2-Gal4; UAS-myr::GCaMP5) were left to freely crawl between a microscope slide and cover slip loosely suspended with tap water. For analysis, x2 binned video data (42 Hz) was further down-sampled by a factor of 2 in the image plane and a factor of 4 in time. Only the green channel was analysed. Following background subtraction, regions of interest were placed as indicated (Fig 3J). Next, from each image frame, we subtracted the mean image of four preceding frames to generate a “running average time-differential” stack—shown as the space–time plot in Fig 3L with the original x-axis collapsed. Individual noncollapsed frames of this stack, separated by 100-ms intervals, are shown in Fig 3M.

### Zebrafish ChR2 activation

A 3 *dpf* zebrafish larva (*Et(E1b*:*Gal4)s1101t*, *Tg(UAS*:*Cr*.*ChR2_H134R-mCherry)s1985t*, *nacre-/-*) was mounted in a drop of E3 medium (5 mM NaCl, 0.17 mM KCl, 0.33 mM CaCl_2_, 0.33 mM MgSO_4_, pH adjusted to 7.4 using NaHCO_3_) on top of a microscope slide and placed immediately above the inverted camera objective. The NeoPixel 12 LED ring was placed about 2 cm above the specimen, facing down. Concurrent maximal activation of all 12 blue LEDs for more than 500 ms reliably elicited pectoral fin swimming bouts. Shorter stimuli were not effective. RGB image data was obtained at 42 Hz, down-sampled by a factor of 4 in time and visualised by tracking the mean brightness of two regions of interest placed onto the pectoral fins.

### *Drosophila* larva ChR2 activation

First, instar *Drosophila* larvae (elav-GAL4/+; UAS-shibre^ts^, UAS-ChR2/+; UAS-ChR2/+, raised on standard food mixed with 200 μM all-trans retinal as described in [33]) were placed on agarose darkened with Indian ink (1% v/v) within the lid of a 50-ml falcon tube and left to freely crawl. The camera and NeoPixel LED ring were placed about 3 cm above the surface. Concurrent activation of all 12 blue LEDs for 1 s at a time reliably triggered larval contractions. Image data acquired at 42 Hz and saved as 8-bit greyscale. Larval length was quantified manually in ImageJ by measuring the distance between head and tail along the body axis at three time points: t = −1, 0.5, and 5 s relative to the flash (t = 0–1 s). *n* = 12 responses from three animals, error bars in standard deviation.

### *Drosophila* adult Chrimson activation

Adult *Drosophila* (w; +; GMR86A08-Gal4/UAS-CsChrimson raised on standard food mixed with 200 μM all-trans retinal as described in [33]) were fixed to a cover slide by gluing the back of their thorax with nail varnish, with limbs moving freely. The NeoPixel 12 LED ring was positioned around the camera objective about 2 cm above the fly, pointing down. Concurrent maximal activation of all 12 red LEDs for 1 s, separated by 2-s intervals, reliably elicited the PER. RGB image data was obtained at 42 Hz (x2 binning). The image stack was converted to 8-bit greyscale, and background over time was subtracted from the entire image stack to limit the excitation light artefact. To calculate proboscis position over time, we plot image brightness over time within a region of interest placed at the tip of the fully extended proboscis.

### Thermogenetics

To assess the performance and stability of the Peltier-Thermistor loop we exported the Peltier command setting and Thermistor reading at 2 Hz through the serial port into an Ascii file and analysed the data using Igor Pro 6 (Wavemetrics).

## Supporting information

S1 Fig Graphical User Interface (GUI). Screenshots of the Python-based GUI divided into four main control panels that can be individually activated depending on user requirements: **A**, Camera control, **B**, LED, **C**, Peltier and Focus Servo control, **D**, Custom protocol window. For details, please refer to the user and assembly manual online: https://github.com/amchagas/Flypi/blob/master/User%20and%20Assembly%20Manual.pdf.
(PDF)

S1 Table Bill of materials (BOM). Complete list, estimated costs and online links to all required parts, organised by modules. For details, please refer to the user and assembly manual online: https://github.com/amchagas/Flypi/blob/master/User%20and%20Assembly%20Manual.pdf.
(XLSX)

S1 Video Zebrafish larva transmission to visualise circulation (related to Fig 2F). (AVI)

S2 Video Zebrafish larva fluorescence sorting (related to Fig 3I). (AVI)

S3 Video Zebrafish larva expressing GFP in the heart (related to Fig 3). (AVI)

S4 Video Zebrafish eggs expressing GCaMP5 in all neurons (related to Fig 3). (AVI)

S5 Video *Drosophila* larva calcium imaging (related to Fig 3J–3M). (AVI)

S6 Video *C*. *elegans* crawling freely (related to Fig 4B). (AVI)

S7 Video *Drosophila* adults walking freely in custom chamber (related to Fig 4D). (AVI)

S8 Video Zebrafish expressing ChR2 in all neurons under blue light (related to Fig 5C and 5D). (AVI)

S9 Video *Drosophila* larvae ChR2 under blue light (related to Fig 5E and 5F). (AVI)

S10 Video *Drosophila* adult proboscis extension reflex driven by CsChrimson using red light (related to Fig 5G and 5H). (AVI)

## Abbreviations

AVI: audio video interleave
BOM: bill of materials
CCD: charge-coupled device
CPU: central processing unit
*dpf*: days postfertilisation
ERG: electroretinogram
GFP: Green Fluorescent Protein
GPP: General Purpose Pin
GUI: Graphical User Interface
IDE: Integrated Development Environment
LED, NO-IR: no infrared [filter]; light-emitting diode
NO-IR: no-infrared filter
PCB: printed circuit board
PER: proboscis extension reflex
*psf*: point-spread function
RGB: red-green-blue
RPi: Raspberry Pi
RPi3: Raspberry Pi 3 single-board computer
SD: secure digital
