# Supplementary material for: The €100 lab: A 3D-printable open-source platform for fluorescence microscopy, optogenetics, and accurate temperature control during behaviour of zebrafish, Drosophila, and Caenorhabditis elegans
Source: PLoS Biol. 2017 Jul 18;15(7):e2002702. doi: 10.1371/journal.pbio.2002702 (PMC5515398; doi:10.1371/journal.pbio.2002702)

Supplementary Figure 1 - Graphical User Interface

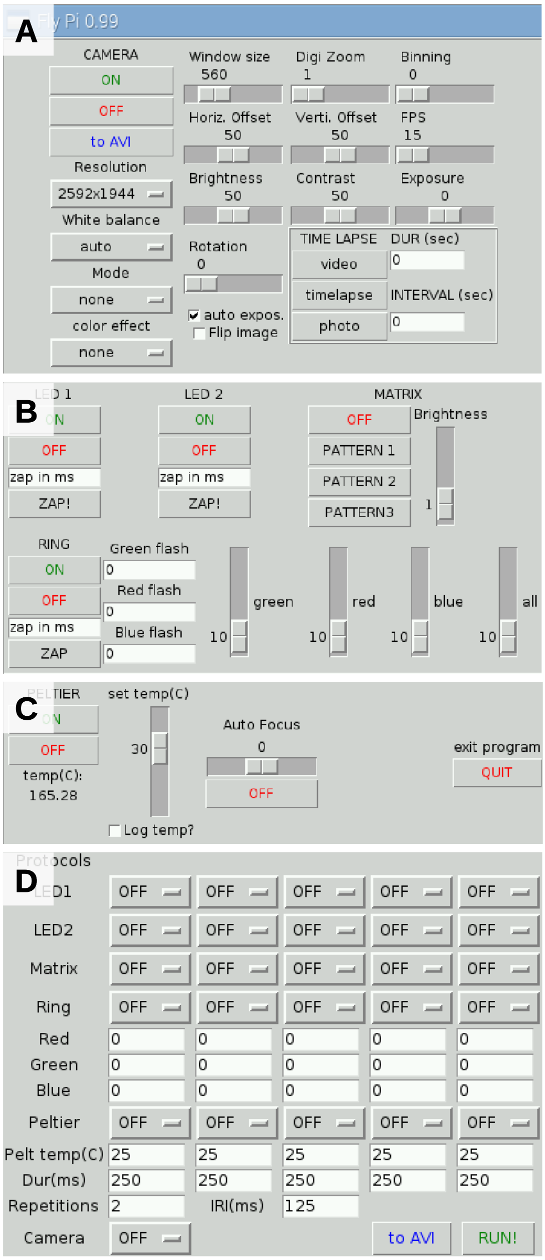

Supplement: S1 Fig — Screenshots of the Python-based GUI divided into four main control panels that can be individually activated depending on user requirements: A, Camera control, B, LED, C, Peltier and Focus Servo control, D, Custom protocol window. For details, please refer to the user and assembly manual online: https://github.com/amchagas/Flypi/blob/master/User%20and%20Assembly%20Manual.pdf. (PDF) [file pbio.2002702.s001.pdf]
